# A model for predicting postoperative persistent acute kidney injury (AKI) in AKI after cardiac surgery patients with normal baseline renal function

**DOI:** 10.1002/clc.24168

**Published:** 2023-10-08

**Authors:** Yuanhan Chen, Zhiming Mo, Hong Chu, Penghua Hu, Wei Fan, Yanhua Wu, Li Song, Li Zhang, Zhilian Li, Shuangxin Liu, Zhiming Ye, Xinling Liang

**Affiliations:** ^1^ Department of Nephrology Guangdong Provincial People's Hospital (Guangdong Academy of Medical Sciences), Southern Medical University Guangzhou China; ^2^ Division of Nephrology The Affiliated Yixing Hospital of Jiangsu University Yixing Jiangsu China

**Keywords:** cardiac surgery, normal renal function, persistent acute kidney injury, precision medicine

## Abstract

**Background:**

Persistent acute kidney injury (AKI) after cardiac surgery is not uncommon and linked to poor outcomes.

**Hypothesis:**

The purpose was to develop a model for predicting postoperative persistent AKI in patients with normal baseline renal function who experienced AKI after cardiac surgery.

**Methods:**

Data from 5368 patients with normal renal function at baseline who experienced AKI after cardiopulmonary bypass cardiac surgery in our hospital were retrospectively evaluated. Among them, 3768 patients were randomly assigned to develop the model, while the remaining patients were used to validate the model. The new model was developed using logistic regression with variables selected using least absolute shrinkage and selection operator regression.

**Results:**

The incidence of persistent AKI was 50.6% in the development group. Nine variables were selected for the model, including age, hypertension, diabetes, coronary heart disease, cardiopulmonary bypass time, AKI stage at initial diagnosis after cardiac surgery, postoperative serum magnesium level of <0.8 mmol/L, postoperative duration of mechanical ventilation, and postoperative intra‐aortic balloon pump use. The model's performance was good in the validation group. The area under the receiver operating characteristic curve was 0.761 (95% confidence interval: 0.737–0.784). Observations and predictions from the model agreed well in the calibration plot. The model was also clinically useful based on decision curve analysis.

**Conclusions:**

It is feasible by using the model to identify persistent AKI after cardiac surgery in patients with normal baseline renal function who experienced postoperative AKI, which may aid in patient stratification and individualized precision treatment strategy.

## Graphical abstract

Among 5368 patients with baseline normal renal function and acute kidney injury (AKI) after cardiac surgery, 3768 patients were randomly used for model development and the rest for validation. We developed a model for predicting postoperative persistent AKI. The model's performance was good in the validation group.

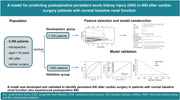

## 1 INTRODUCTION

Acute kidney injury (AKI) is a serious and major complication of cardiac surgery.^1^, ^2^ Even for patients with normal renal function before cardiac surgery, AKI is not uncommon with a prevalence ranging from 14.6% to 43.0%.^3^, ^4^ For most patients with AKI, kidney dysfunction could be rapidly relieved with the improvement in the acute illness in the short term. However, renal insufficiency fails to recover in up to 44.7% of postoperative AKI patients undergoing cardiac surgery.^5^ Renal recovery following AKI has been largely neglected and no standardized management consensus existed. To draw the clinicians’ attention to renal recovery after renal injury and to its standardized management, the Acute Dialysis Quality Initiative (ADQI) introduced the concept of persistent AKI defined as the duration of AKI longer than 2 days.^6^ Compared to AKI patients whose renal dysfunction was relieved quickly, patients with persistent AKI have higher in‐hospital mortality, are more likely to develop chronic kidney disease, and have worse long‐term prognosis.^7^ Due to limited effective therapeutic and treatment strategies, tools for early identification of high‐risk persistent AKI groups are needed for early intervention and to improve prognosis.

Novel biomarkers may aid in identifying persistent AKI.^8^ However, patients included in previous studies have experienced moderate to severe AKI. The performance of those biomarkers for predicting persistent AKI in patients with mild AKI is unknown. In addition, biomarker use requires specialized and costly instruments, limiting their application in routine clinical practice, especially in less developed areas. With the increasing availability of electronic medical record data, using such data to develop a model may aid in clinicians’ timely assessment of the risk of persistent AKI.

At present, several risk prediction models for persistent AKI have been introduced.^9^ Unfortunately, few data from patients with cardiac surgery were evaluated in previous models. Due to the heterogeneity of the disease, previous models may not be applicable for patients undergoing cardiac surgery. A number of predisposing risk factors for persistent AKI after cardiac surgery have been identified.^10^, ^11^ However, most previous studies have not integrated early biochemical and clinical data in an attempt to forecast persistent AKI after cardiac surgery. Therefore, we develop and validate a predictive model for persistent AKI after cardiac surgery in patients with normal baseline renal function who experienced AKI after cardiac surgery.

## 2 METHODS

### 2.1 Population and study protocol

Due to the nature of the present single‐center, retrospectively designed study, the need for signed informed consent from participants was waived. Data were analyzed anonymously. The Ethics Committee of Guangdong Provincial People's Hospital approved the study according to the Declaration of Helsinki (No. GDREC2018416H).

The data from patients with normal renal function at baseline who experienced AKI after cardiac surgery requiring cardiopulmonary bypass between January 1, 2006 and December 31, 2018 at the Guangdong Provincial People's Hospital (a tertiary teaching hospital) were retrospectively analyzed. There were the following exclusion criteria: renal replacement therapy before surgery; history of unilateral nephrectomy; cardiac transplantation; emergency surgery; critical status before surgery; participants hospitalized for a maximum of 2 days after the AKI episode; and serum creatinine values were not available within 1 week after the AKI episode. The estimated glomerular filtration rate (eGFR) greater than or equal to 60 mL/min × 1.72 m^2^ was considered normal renal function. The eGFR was assessed using the Chronic Kidney Disease‐Epidemiology Collaboration formula utilizing baseline serum creatinine.^12^ The lowest value 3 months before admission was defined as the baseline serum creatinine. If preadmission values were not available, the minimum preoperative creatinine value during hospitalization was used. The AKI was diagnosed using the creatinine criteria outlined by the Kidney Disease: Improving Global Outcomes (KDIGO).^13^ The critical condition was referred to the definition of the European System for Cardiac Operative Risk Evaluation II.^14^ For patients with multiple cardiac surgeries between January 1, 2006 and December 31, 2018, data of the first cardiac surgery during the study period were analyzed.

### 2.2 Data collection and definition

The data for patients were extracted from the electronic medical record system of our hospital. Based on previous literature review and clinical expertise, the following candidate predictors were considered: demographic characteristics, comorbidities (hypertension, diabetes, and coronary atherosclerotic heart disease), history of heart surgery, baseline serum creatinine level, preoperative left ventricular ejection fraction (LVEF), cardiac surgery type, cardiopulmonary bypass time, AKI stage at the initial diagnosis of AKI after cardiac surgery, postoperative medication use, laboratory test results after surgery, duration of postoperative mechanical ventilation, postoperative intra‐aortic balloon pump (IABP) use, and reoperation status. AKI stage at the initial diagnosis of AKI after cardiac surgery was classified by the KDIGO criteria upon first AKI diagnosis after surgery. Postoperative variable data, such as postoperative medication use, laboratory test results after surgery, and reoperation status, were collected from the time after cardiac surgery to the time at first diagnosis of postoperative AKI. If more than one laboratory test result was available after surgery, the latest result before the time of the initial diagnosis of AKI after cardiac surgery was analyzed. Previous cardiac surgery was defined as the cardiac surgery was underwent before January 1, 2006.

### 2.3 Outcome

The outcome of interest was persistent AKI. It was defined according to the consensus report for the ADQI, where the duration of AKI defined by the KDIGO criteria was longer than 2 days.^6^, ^13^

### 2.4 Sample size

The sample size for developing a predictive model was determined by events per variable. Empirically, a minimum of 10 events per variable is generally needed.^15^ At least 340 events are needed for developing a model with 34 potential predictors. Assuming that the incidence of persistent AKI was 50% according to our previous reports, more than 680 patients for developing a model is reasonable.

### 2.5 Statistical analysis

Patients were randomly assigned to the development and validation groups at a ratio of 7:3. The clinical expertise and boxplot plots were used to check for extreme values in the continuous variables. Then, continuous variables (preoperative LVEF, baseline eGFR, cardiopulmonary bypass time, postoperative hemoglobin level, postoperative white blood cell count, postoperative serum magnesium level, postoperative carbon dioxide combining power, and postoperative serum uric acid level) were winsorized at 1% and 99% to reduce the effect of the extreme values. Multiple imputation by chained equations with 20 iterations was used to estimate the missing data, and the results were pooled according to the Rubin's rule. All variables, including the outcome variable, were included in the imputation model, which was recommended for handling missing data.^16^ Continuous variables were reported as medians and interquartile ranges or means and standard deviations, while categorical variables were represented as frequencies (percentages). Mann–Whitney *U* test or *t*‐test was utilized for differences between groups of continuous variables, and the *χ* ^2^ test or Fisher's exact test was applied for categorical variables. Compared to serum creatinine levels, eGFR was more suitable for evaluating renal function.^17^ Therefore, eGFR was used for developing the model to avoid multicollinearity and improve its performance. Collinearity between variables was examined using variance inflation factors. Variance inflation factors less than five indicated a weak collinearity.

Restricted cubic splines were used to analyze the potential nonlinear association between continuous variables and the outcome risk.^18^ If a nonlinear relationship was identified, a continuous variable was converted to a categorical variable for analysis on the basis of previous literature and clinical expertise. The model was developed using logistic regression analysis with predictors selected through least absolute shrinkage and selection operator (LASSO) regression in the development group. LASSO used L1 penalty, which is effective in addressing multicollinearity and limiting overfitting.^19^ To avoid overfitting, a 10‐fold cross‐validation and a one standard error rule were applied. A nomogram was generated according to the weight of each predictor in the final model to make it easier use in clinical practice.

The final model was validated in the development and validation groups, respectively. The performance of the model was assessed by its discrimination, calibration, and clinical utility. The area under the receiver operating characteristic curve (AUC) was used to evaluate the discrimination. In general, the AUC > 0.7 indicated a good discrimination. The calibration of the model was assessed by the calibration curve and examined utilizing the Spiegelhalter *Z* test.^20^ When the predicted probability is exactly the same as the actual probability, the calibration curve coincides with the 45° diagonal line, where the slope is 1 and the intercept is equal to 0. Decision curve analysis (DCA) was conducted by quantifying the net benefits with a series of threshold probabilities and its results were utilized to assess the clinical utility in the validation group.^21^

All analyses results for developing and validating the model were in accordance with the Transparent Reporting of a multivariable prediction model for Individual Prognosis Or Diagnosis guidelines. R software (version 4.2.1; https://www.r-project.org) and IBM SPSS v.25.0 (SPSS IBM) were used to analyze the data. A *p* value of <.05 indicated a statistically significant result.

## 3 RESULTS

A total of 6106 patients were identified, and 5368 were ultimately included in the present study. The data for 3768 patients were randomly assigned to develop the model, while the data for the remaining 1600 patients were used for validating the model. A summary of the screening results is presented (Supporting Information: Figure S1). There were 10 variables with missing data in both development and validation groups. Distribution and percentage of missing data for each variable in development and validation groups are shown in Supporting Information: Table S1. The incidence of persistent AKI was 50.6% (*n* = 1905) and 48.5% (*n* = 776) in the development and validation groups, respectively. The baseline clinical characteristics of the development and validation sets are shown in Table 1.

**Table 1:** Baseline characteristics.

Variables	Development group (*n* = 3768)	Validation group (*n* = 1600)
Age, years	55.0 (46.0, 62.0)	55.0 (45.0, 63.0)
Male, *n* (%)	2184 (58.0)	924 (57.8)
Baseline serum creatinine, µmol/L	78.0 (65.0, 89.0)	78.4 (65.0, 89.2)
eGFR, mL/min/1.73 m^2^	88.9 (75.3, 101.3)	88.3 (74.3, 101.0)
LVEF, %	60.0 (55.0, 65.0)	60.0 (55.0, 64.0)
Comorbidities, *n* (%)		
Hypertension	1139 (30.2)	527 (32.9)
Diabetes mellitus	286 (7.6)	128 (8.0)
Coronary heart disease	574 (15.2)	255 (15.9)
COPD	76 (2.0)	32 (2.0)
Infectious endocarditis	218 (5.8)	87 (5.4)
Cerebrovascular disease	216 (5.7)	95 (5.9)
Peripheral vascular disease	23 (0.6)	11 (0.7)
Atrial fibrillation	1132 (30.0)	483 (30.2)
PCI history, *n* (%)	62 (1.6)	24 (1.5)
Previous cardiac surgery, *n* (%)	134 (3.6)	68 (4.2)
History of transfusion, *n* (%)	15 (0.4)	2 (0.1)
Procedure, *n* (%)		
On‐pump CABG	237 (6.3)	108 (6.8)
Valve	2462 (65.3)	1038 (64.9)
Aortic	454 (12.0)	180 (11.2)
CHD	359 (9.5)	159 (9.9)
On‐pump CABG + valve	225 (6.0)	103 (6.4)
Others	31 (0.8)	12 (0.8)
CPB time, min	130.0 (130.0, 141.0)	130.0 (130.0, 144.0)
AKI stage at initial diagnosis after cardiac surgery, *n* (%)		
1	3225 (85.6)	1378 (86.1)
2	505 (13.4)	207 (12.9)
3	38 (1.0)	15 (0.9)
Postoperative laboratory findings		
Hemoglobin, g/L	111.0 (101.0, 120.3)	111.7 (101.8, 120.8)
Platelet, ×10^9^/L	130.0 (97.0, 162.0)	128.0 (100.0, 164.0)
Blood leukocytes, ×10^9^/L	7.9 (5.6, 10.3)	8.2 (5.8, 10.5)
Natremia, mmol/L	143.7 (141.1, 145.6)	144.0 (141.0, 145.7)
Potassium, mmol/L	4.3 (4.0, 4.5)	4.3 (4.0, 4.5)
Magnesemia, mmol/L	1.1 (0.9, 1.2)	1.1 (0.9, 1.2)
CO_2_CP, mmol/L	24.9 (23.0, 26.5)	24.9 (23.0, 26.5)
Uric acid, μmol/L	452.0 (390.0, 521.0)	461.0 (390.0, 524.0)
Postoperative durgs use, *n* (%)		
RASIs	374 (9.9)	176 (11.0)
NSAID	68 (1.8)	33 (2.1)
Aminoglycoside antibiotics	69 (1.8)	31 (1.9)
Statin	62 (1.6)	31 (1.9)
Proton pump inhibitors	1395 (37.0)	566 (35.4)
Postoperative MV duration, h	16.0 (3.0, 32.0)	15.0 (3.0, 30.0)
Postoperative IABP, *n* (%)	180 (4.8)	60 (3.8)
Reoperation, *n* (%)	223 (5.9)	93 (5.8)

*Note*: Data of all variables were obtained before or at the initial diagnosis of AKI after cardiac surgery. Values are expressed as medians and interquartile ranges unless otherwise noted.

Abbreviations: AKI, acute kidney injury; CABG, coronary artery bypass grafting; CHD, congenital heart disease; CO_2_CP, carbon dioxide combining power; COPD, chronic obstructive pulmonary disease; CPB, cardiopulmonary bypass; eGFR, estimated glomerular filtration rate; IABP, intra‐aortic balloon pump; LVEF, left ventricular ejection fraction; MV, mechanical ventilation; NSAID, nonsteroidal anti‐inflammatory drug; PCI, percutaneous coronary intervention; RASIs, renin–angiotensin system inhibitors.

**Figure 1:**
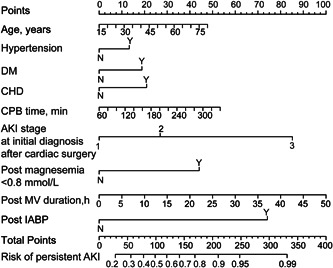
Nomogram for predicting persistent AKI after cardiac surgery. AKI, acute kidney injury; CHD, congenital heart disease; CPB, cardiopulmonary bypass; DM, diabetes mellitus; IABP, intra‐aortic balloon pump; MV, mechanical ventilation.

Patients were divided into two groups according to whether persistent AKI occurred. All predictors were compared in the development group. Some variables, such as male gender, hypertension, and postoperative duration of mechanical ventilation, may be potential risk factors for persistent AKI (Supporting Information: Table S2). To develop the model, a total of 34 variables as shown in Supporting Information: Table S2 are included in the LASSO regression. Then, nine variables with non‐zero coefficients were selected as the predictors of persistent AKI, including age, hypertension, diabetes, coronary heart disease, cardiopulmonary bypass time, AKI stage at initial diagnosis after cardiac surgery, postoperative serum magnesium level of <0.8 mmol/L, postoperative duration of mechanical ventilation, and postoperative IABP use (Supporting Information: Figure S2). Finally, the new model was developed by using the logistic regression analysis with the above variables. The regression coefficients for each variable are presented in Table 2. A nomogram was generated to visualize the model (Figure 1).

**Table 2:** Multivariate logistic regression analysis of variables for predicting persistent AKI after cardiac surgery.

Variables	*β*	SE	*p*	OR	95% CI
Age, years	0.015	0.003	<0.001	1.015	(1.008, 1.021)
Comorbidities					
Hypertension	0.262	0.083	0.002	1.300	(1.104, 1.529)
Diabetes mellitus	0.372	0.150	0.013	1.450	(1.080, 1.947)
Coronary heart disease	0.413	0.113	<0.001	1.511	(1.211, 1.886)
CPB time, min	0.004	0.001	<0.001	1.004	(1.002, 1.006)
AKI stage at initial diagnosis after cardiac surgery			<0.001		
1				1.000	
2	0.531	0.109	<0.001	1.700	(1.373, 2.106)
3	1.682	0.517	0.001	5.378	(1.952, 14.814)
Postoperative magnesemia <0.8 mmol/L	0.871	0.130	<0.001	2.390	(1.851, 3.085)
Postoperative MV duration	0.039	0.002	<0.001	1.040	(1.035, 1.045)
Postoperative IABP	1.465	0.251	<0.001	4.326	(2.645, 7.075)
Constant	−2.393	0.220	<0.001		

*Note*: Data of all variables were obtained before or at the initial diagnosis of AKI after cardiac surgery.

Abbreviations: AKI, acute kidney injury; CI, confidence interval; CPB, cardiopulmonary bypass; IABP, intra‐aortic balloon pump; MV, mechanical ventilation; OR, odds ratio; SE, standard error.

**Figure 2:**
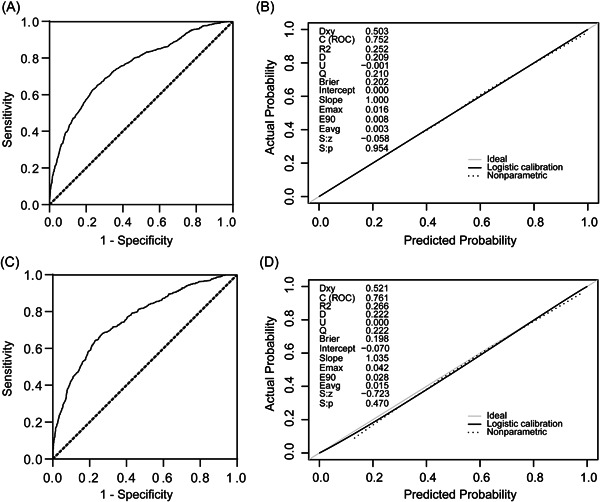
Model performance was evaluated using receiver‐operating characteristic and calibration curves. (A) AUC for the model in the development group showing a mean AUC of 0.752 (95% CI: 0.736–0.767). (B) Calibration curve for the new model in the development group. (C) AUC for the model in the validation group showing a mean AUC of 0.761 (95% CI: 0.737–0.784). (D) Calibration curve for the new model in the validation group. Calibration plots show the relationship between the predicted values and actual incidence of persistent AKI. Solid and dotted lines are closely matched, which indicates a more accurate prediction model. AKI, acute kidney injury; AUC, area under the receiver operating characteristic curve.

**Figure 3:**
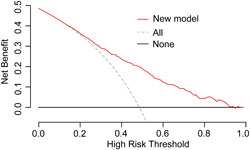
Clinical values for the prediction model with decision curve analyses (DCA). Threshold probability was represented versus the net benefit. Dashed and solid black lines indicated the hypothesis of “all patients” and “no patients with persistent AKI,” respectively. As the curve on the DCA graph approaches the top, the model diagnosis value increases. AKI, acute kidney injury.

The discrimination and calibration of the model in the development group (Figure 2A,B). The AUC was 0.752 (95% confidence interval [CI]: 0.736–0.767). To increase the reliability of its extrapolation, the model was also evaluated in the validation group. The model's performance did not significantly change. The AUC was 0.761 (95% CI: 0.737–0.784). The predicted values were in good agreement with the actual values, as presented in the calibration curve (Figure 2C,D). To assess clinical usefulness, the DCA curve indicated that the net benefit was obtained using the new model across the majority of the threshold probability range (Figure 3).

## 4 DISCUSSION

A model for predicting persistent AKI after cardiac surgery in patients with normal baseline renal function who experienced AKI after cardiac surgery was developed using routinely collected clinical data. The model was also evaluated based on its discrimination, calibration, and clinical utility in the validation group. To the best of our knowledge, this is the first study to develop and validate a model for predicting persistent AKI after cardiac surgery. The new model may aid the reevaluation and clinical management of patients with AKI after cardiac surgery to reduce the chance of chronic kidney disease and improve prognosis.

Some scholars call for clinicians to pay more attention to persistent AKI, since it is more consistent with what clinicians understand as the true “clinical AKI”.^22^ Early identification of persistent AKI is conducive to its re‐evaluation and clinical management re‐optimization to prevent or alleviate further progression of the injury. The present model for predicting persistent AKI after cardiac surgery may help patient stratification and individualized precision treatment strategy. For high‐risk patients, large fluid infusions may be limited to avoid fluid overload. Hemodynamic monitoring, renal function monitoring, and nephrology consultation might be emphasized to reduce the risk of chronic kidney disease. Meanwhile, for low‐risk populations, the watchful waiting strategy and reducing the frequency of renal function monitoring may be reasonable. In addition, early identification of persistent AKI may be helpful to determine the timing of renal replacement therapy, facilitating rational and effective allocation of medical resources. Early initiation of renal replacement therapy may be considered in patients without urgent dialysis indications but with a high risk of persistent AKI. Conversely, early initiation of renal replacement therapy might be postponed unless the absolute medical indication was present in patients at low risk for persistent AKI. Finally, prompt identification of persons with a high risk for developing persistent AKI using the new model could be beneficial for appropriate research subject enrollment in clinical trials to assess the effectiveness of the strategies.

Limited data on the rate of persistent AKI after cardiac surgery were reported in previous studies. The occurrence rate of persistent AKI fluctuated from 10.4% to 71.4% in limited number of previous studies due to different diagnostic criteria and study populations.^11^, ^23^, ^24^ The 16th ADQI Working group recommended the definition of persistent AKI to identify high‐risk patients for additional examination and evaluation. Similar to a previous prospective study where the incidence of persistent AKI after cardiac surgery defined according to the ADQI criteria was 52%,^25^ the incidence in the present study was 50.6%.

Several predictors, such as age, hypertension, diabetes, coronary heart disease, postoperative IABP use, and AKI stage at the initial diagnosis of AKI, were risk factors for persistent AKI in previous reports.^10^, ^11^, ^26^ These variables were also predictors in our model. In addition, cardiopulmonary bypass time, postoperative duration of mechanical ventilation, and postoperative serum magnesium level were also predictors in our model. They were risk factors for AKI in previous findings.^27^, ^28^ However, data on the association of cardiopulmonary bypass time, postoperative duration of mechanical ventilation, and postoperative serum magnesium level with postoperative persistent AKI were limited. In a prospective study enrolling 3245 patients who underwent cardiac surgery and with a follow‐up of 2 years, the long duration of cardiopulmonary bypass was a risk factor for chronic kidney disease.^29^ Moreover, as a retrospective study indicated that mechanical ventilation may be a predictor for persistent AKI in critical patients. Patients utilizing mechanical ventilation had a 61% increased risk of persistent AKI.^30^ Finally, hypomagnesemia had an adverse effect on the restoration of renal function after injury.^31^ Hence, it is plausible that these variables may predict persistent AKI.

Admittedly, the present study had some limitations. First, even though the sample size was relatively large, the model needs to be verified at other independent centers since it was a single‐center study. Second, data were missing for some variables. Multiple imputation was used to minimize the effect of missing data on model performance as recommended by previous reports. Finally, data for some variables, such as proteinuria and hemodynamic variables before or at the initial diagnosis of AKI after cardiac surgery, were not obtained due to the retrospective nature of the study. The influence of these variables on the model's performance was unknown. For the same reason, aortic cross‐clamp time, surgical anesthesia duration, and intraoperative use of red blood cells and platelets were not analyzed. Fortunately, the model developed using the available data showed a good performance.

## 5 CONCLUSION

In the present study, a model for predicting persistent postoperative AKI in patients with normal baseline renal function who experienced AKI after cardiac surgery was developed and validated. This model can identify population at high risk of persistent AKI after cardiac surgery early, facilitate the re‐evaluation of medical regimen to avoid further damage after kidney injury, and provide individualized treatment strategies. In the future, efforts will be made to integrate the model into the existing electronic medical records system to make its clinical use more convenient.

## AUTHOR CONTRIBUTIONS

*Conceptualization*: Yuanhan Chen, Penghua Hu, and Xinling Liang. *Methodology*: Yuanhan Chen, Penghua Hu, Zhiming Mo, and Hong Chu. *Software*: Yuanhan Chen and Penghua Hu. *Validation*: Wei Fan, Yanhua Wu, and Li Song. *Formal analysis*: Yuanhan Chen, Penghua Hu, and Li Zhang. *Investigation*: Yuanhan Chen, Penghua Hu, Zhiming Mo, and Zhilian Li. *Resources*: Yuanhan Chen and Xinling Liang. *Data curation*: Penghua Hu and Zhiming Mo. *Writing—original draft preparation*: Yuanhan Chen. *Writing—review and editing*: Penghua Hu, Shuangxin Liu, Zhiming Ye, and Xinling Liang. *Visualization:* Yuanhan Chen and Penghua Hu. *Supervision*: Hong Chu and Xinling Liang. *Project administration*: Xinling Liang. *Funding acquisition*: Yuanhan Chen and Xinling Liang. All authors have read and agreed to the published version of the manuscript.

## CONFLICT OF INTEREST STATEMENT

The authors declare no conflict of interest

## Supporting information

Supporting information.

## Data Availability

The datasets used and/or analyzed during the current study are available from the corresponding author on reasonable request. The data are not publicly available due to privacy or ethical restrictions.
