# Supplementary material for: A model for predicting postoperative persistent acute kidney injury (AKI) in AKI after cardiac surgery patients with normal baseline renal function
Source: Clin Cardiol. 2023 Oct 8;47(1):e24168. doi: 10.1002/clc.24168 (PMC10766121; doi:10.1002/clc.24168)
Supplement: Supplementary file 1 — Supporting information. [file CLC-47-e24168-s001.doc]

**Supplemental Figures**

**Figures S1. Flowchart of the participant screening process**. AKI, acute kidney injury; RRT renal replacement therapy.


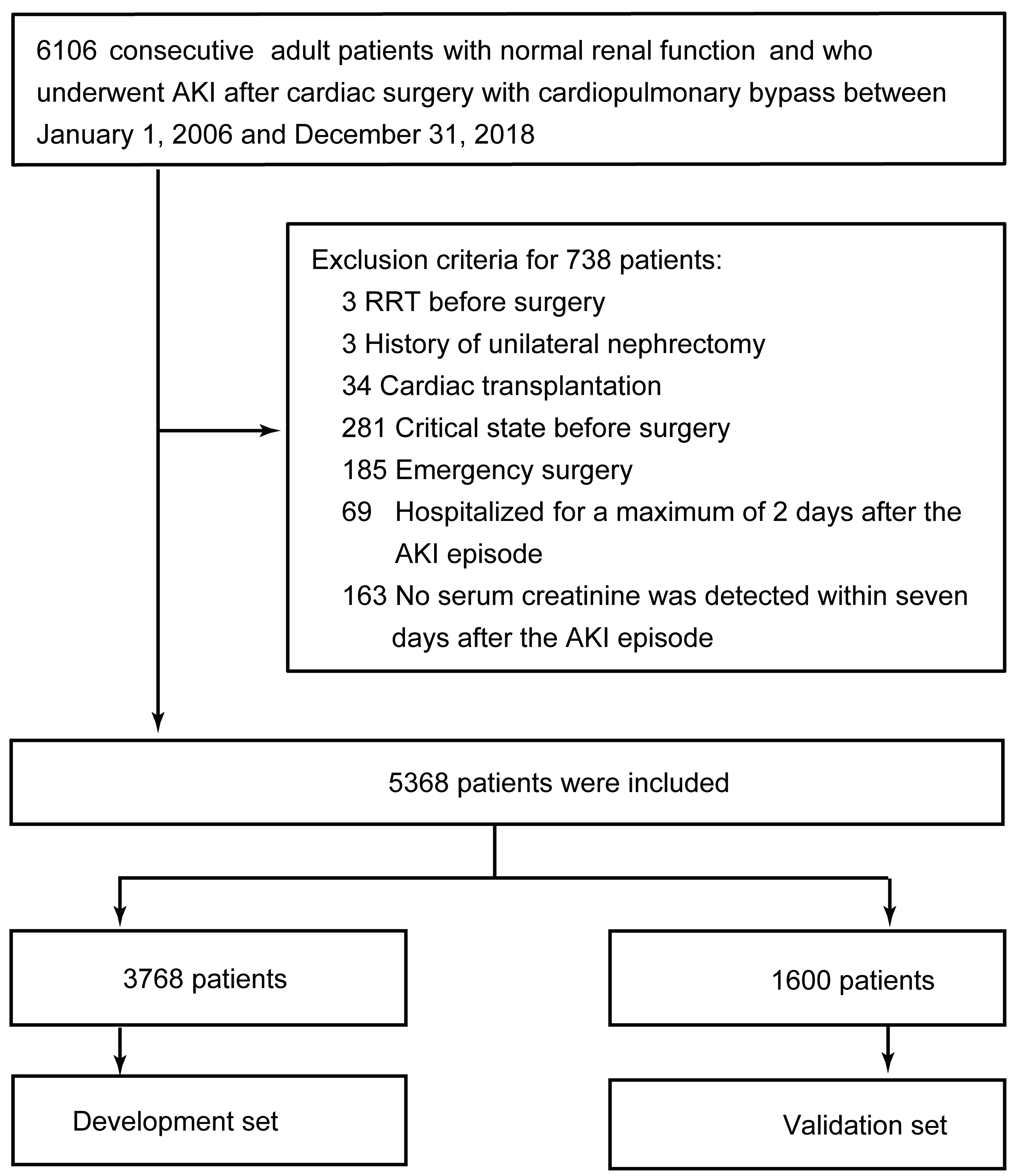


**Figures S2. Variable selection using the LASSO regression method**. (a) Identification of the penalty tuning parameter (λ) in the LASSO model using ten-fold cross-validation and minimum criteria. AUC was plotted vs. log(λ). Dotted vertical lines were plotted at the optimal values with minimum criteria and one standard error of minimum criteria (1-SE criteria). The λ value of 0.0239 and log(λ) of -3.734 were chosen. (b) Coefficient profile plot of 34 predictors. The same criteria as in Figure 1a were used to plot dotted vertical lines at ideal values. A total of nine predictors with coefficients other than zero were selected. *AUC,* area under the receiver operating characteristic curve; *LASSO* least absolute shrinkage and selection operator; *SE,* standard error.


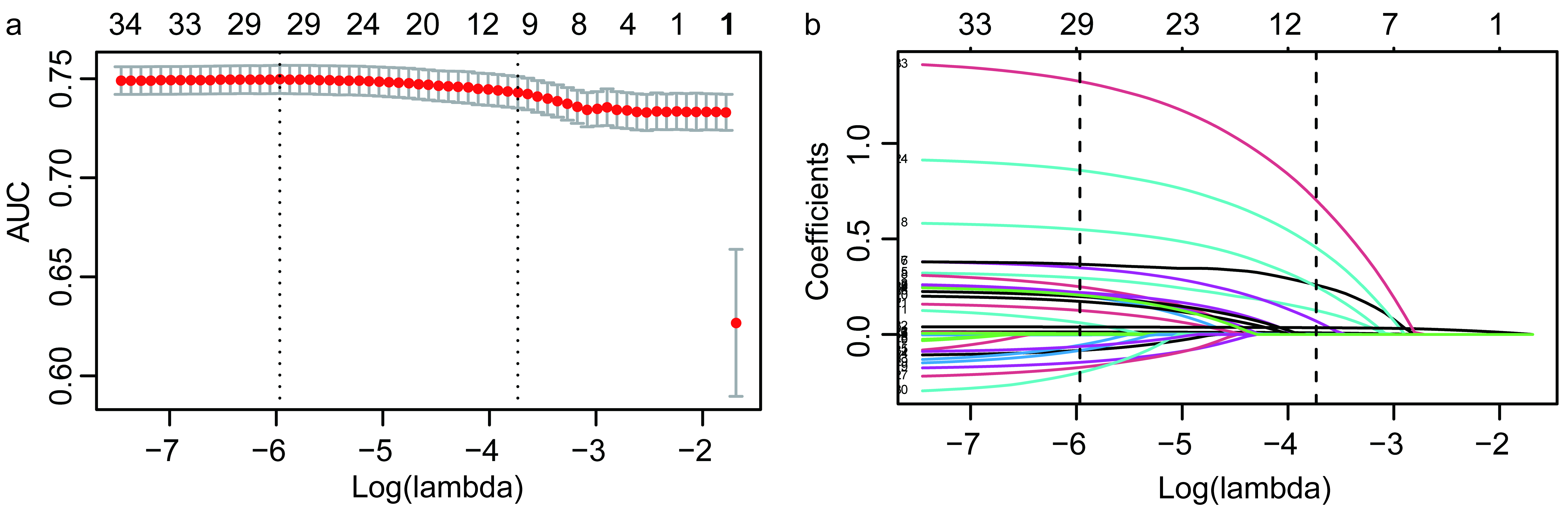


**Supplemental Tables**

**Table [S1](https://www.ncbi.nlm.nih.gov/pmc/articles/PMC10018083/#clc23969-suppl-0001).** Distribution and percentage of missing data for variables in development and validation groups.

| Variables | Development group (n=3768) | | Validation group (n=1600) | |
| --- | --- | --- | --- | --- |
|  | Distribution | Missing, n (%) | Distribution | Missing, n (%) |
| LVEF, % | 61.0 (54.0, 65.0) | 235 (6.2) | 61.0 (54.0, 64.0) | 107 (6.7) |
| CPB time, min | 130.0 (127.0, 148.0) | 480 (12.7) | 130.0 (130.0, 148.0) | 196 (12.3) |
| Postoperative laboratory findings prior to or at first diagnosis of postoperative AKI | | | | |
| Hemoglobin, g/L | 110.2 (100.0, 121.0) | 160 (4.2) | 111.0 (101.0, 121.0) | 68 (4.2) |
| Platelet, ×10^9^/L | 127.3 (96.0, 164.0) | 161 (4.3) | 126.0 (98.0, 166.8) | 68 (4.2) |
| Blood leucocytes, ×10^9^/L | 7.7 (5.5, 10.4) | 179 (4.8) | 7.9 (5.7, 10.7) | 77 (4.8) |
| Natremia, mmol/L | 143.3 (140.3, 146.4) | 741 (19.7） | 143.3 (140.2, 146.4) | 306 (19.1) |
| Potassium, mmol/L | 4.3 (3.9, 4.6) | 741 (19.7） | 4.3 (4.0, 4.6) | 306 (19.1) |
| Magnesemia, mmol/L | 1.0 (0.9, 1.2) | 720 (19.1) | 1.0 (0.9, 1.2) | 304 (19.0) |
| CO2CP, mmol/L | 24.8 (22.4, 27.2) | 750 (19.9) | 24.9 (22.3, 27.1) | 309 (19.3) |
| Uric acid, μmol/L | 456.0 (369.5, 541.0) | 681 (18.1) | 458.0 (362.0, 543.0) | 300 (18.8) |

Note: Values are expressed as medians and interquartile ranges.

Abbreviations: *AKI, acute kidney injury; CO2CP,* carbon dioxide combining power; *CPB,* cardiopulmonary bypass; *LVEF,* left ventricular ejection fraction.

**Table S2.** Univariate analysis of candidate variables and persistent AKI in the development group.

| Variables | None persistent AKI (n=1863) | Persistent AKI (n=1905) | *P* value |
| --- | --- | --- | --- |
| Age, years | 53.0 (43.0, 60.0) | 57.0 (48.0, 64.0) | <0.001 |
| Male, n (%) | 1019 (54.7%) | 1165 (61.2%) | <0.001 |
| eGFR, ml/min/1.73m^2^ | 90.9 (77.0, 103.3) | 86.7 (74.1, 99.1) | <0.001 |
| LVEF, % | 61.0 (56.0, 65.0) | 60.0 (54.0, 64.0) | 0.001 |
| Comorbidities, n (%) |  |  |  |
| Hypertension | 431 (23.1%) | 708 (37.2%) | <0.001 |
| Diabetes mellitus | 90 (4.8%) | 196 (10.3%) | <0.001 |
| Coronary heart disease | 192 (10.3%) | 382 (20.1%) | <0.001 |
| COPD | 27 (1.4%) | 49 (2.6%) | 0.014 |
| Infectious endocarditis | 99 (5.3%) | 119 (6.2%) | 0.220 |
| Cerebrovascular disease | 82 (4.4%) | 134 (7.0%) | 0.001 |
| Peripheral vascular disease | 8 (0.4%) | 15 (0.8%) | 0.158 |
| Atrial fibrillation | 575 (30.9%) | 557 (29.2%) | 0.277 |
| PCI history, n (%) | 15 (0.8%) | 47 (2.5%) | <0.001 |
| Previous cardiac surgery, n (%) | 64 (3.4%) | 70 (3.7%) | 0.692 |
| History of transfusion, n (%) | 9 (0.5%) | 6 (0.3%) | 0.413 |
| Procedure, n (%) |  |  | <0.001 |
| on-pump CABG | 81 (4.3%) | 156 (8.2%) |  |
| Valve | 1332 (71.5%) | 1130 (59.3%) |  |
| Aortic | 138 (7.4%) | 316 (16.6%) |  |
| CHD | 223 (12.0%) | 136 (7.1%) |  |
| on-pump CABG + valve | 69 (3.7%) | 156 (8.2%) |  |
| Others | 20 (1.1%) | 11 (0.6%) |  |
| CPB time, min | 130.0 (123.0, 141.0) | 130.0 (130.0, 150.0) | <0.001 |
| AKI stage at initial diagnosis after cardiac surgery, n (%) | |  | <0.001 |
| 1 | 1660 (89.1%) | 1565 (82.2%) |  |
| 2 | 198 (10.6%) | 307 (16.1%) |  |
| 3 | 5 (0.3%) | 33 (1.7%) |  |
| Postoperative laboratory findings | | | |
| Hemoglobin<120 g/L, n (%) | 1356 (72.8%) | 1422 (74.6%) | 0.195 |
| Platelet<100 ×10^9^/L, n (%) | 432 (23.2%) | 573 (30.1%) | <0.001 |
| Blood leucocytes≧ 10×10^9^/L, n (%) | 502 (26.9%) | 529 (27.8%) | 0.571 |
| Natremia, mmol/L | 143.7 (140.7, 145.4) | 143.7 (141.6, 145.7) | <0.001 |
| Potassium <3.5 mmol/L, n (%) | 67 (3.6%) | 83 (4.4%) | 0.232 |
| Magnesemia <0.8 mmol/L, n (%) | 103 (5.5%) | 260 (13.6%) | <0.001 |
| CO2CP ≧28 mmol/L, n (%) | 263 (14.1%) | 324 (17.0%) | 0.014 |
| Uric acid ≧450 μmol/L, n (%) | 1076 (57.8%) | 1204 (63.2%) | 0.001 |
| Postoperative durgs use, n (%) | | | |
| RASIs | 166 (8.9%) | 208 (10.9%) | 0.039 |
| NSAID | 23 (1.2%) | 45 (2.4%) | 0.009 |
| Aminoglycoside antibiotics | 33 (1.8%) | 36 (1.9%) | 0.786 |
| Statin | 23 (1.2%) | 39 (2.0%) | 0.050 |
| Proton pump inhibitors | 609 (32.7%) | 786 (41.3%) | <0.001 |
| Postoperative MV duration, h | 11.0 (0.0, 18.0) | 23.0 (3.0, 48.0) | <0.001 |
| Postoperative IABP, n (%) | 21 (1.1%) | 159 (8.3%) | <0.001 |
| Reoperation, n (%) | 86 (4.6%) | 137 (7.2%) | 0.001 |

Note: Data of all variables were obtained prior to or at the initial diagnosis of AKI after cardiac surgery. Values are expressed as medians and interquartile ranges unless otherwise noted.

Abbreviations: *AKI,* acute kidney injury; *CABG,* coronary artery bypass grafting; *CHD,* congenital heart disease; *CO2CP,* carbon dioxide combining power; *COPD,* chronic obstructive pulmonary disease; *CPB,* cardiopulmonary bypass; *eGFR,* estimated glomerular filtration rate; *IABP,* intra-aortic balloon pump; *LVEF,* left ventricular ejection fraction; *MV,* mechanical ventilation; *NSAID,* non-steroidal anti-inflammatory drug; *PCI,* percutaneous coronary intervention; *RASIs,* renin-angiotensin system inhibitors.
